# Total Corneal Aberration Measurements by Anterior Segment Optical Coherence Tomography and Scheimpflug-Based Imaging: A Narrative Review of Optical Principles and Sources of Inter-Device Discordance

**DOI:** 10.3390/jcm15208174

**Published:** 2026-10-21

**Authors:** Doyeon Kim, Seung Pil Bang

**Affiliations:** 1 Department of Medicine, Keimyung University School of Medicine, Daegu 42601, Republic of Korea; doyeonkim.med@gmail.com; 2 Department of Ophthalmology, Keimyung University School of Medicine, Daegu 42601, Republic of Korea

**Keywords:** total corneal aberration, higher-order aberration, anterior segment optical coherence tomography, Scheimpflug imaging, corneal tomography, wavefront analysis, intraocular lens calculation

## Abstract

Total corneal aberration analysis is increasingly used as part of intraocular lens selection, ectasia screening, and refractive surgery planning. Scheimpflug-based tomography and anterior segment optical coherence tomography (AS-OCT) are commonly used imaging techniques to identify the anterior and posterior corneal surfaces; however, many devices combine these methods with Placido-disc topography or reconstruction algorithms. Although these devices provide anterior, posterior, and total corneal wavefront aberrations, multiple studies have shown that Zernike coefficients obtained with different systems are not consistently interchangeable, particularly for posterior-surface-derived and higher-order terms such as trefoil and total higher-order aberration root-mean-square values. This narrative review summarizes the optical principles of Scheimpflug imaging and AS-OCT, synthesizes the available comparative literature, and analyzes the mechanisms that may contribute to inter-device discordance, including differences in light sources and light-tissue interactions, posterior-surface reconstruction and refractive-index assumptions, edge-detection and Zernike-fitting algorithms, sampling geometry and acquisition time, and reference-axis selection. We discuss the clinical implications of these discrepancies and propose a research agenda, including model-eye validation and ray-tracing simulation studies, to help distinguish the effects of hardware optics from those of proprietary reconstruction algorithms.

## 1. Introduction

Corneal wavefront aberrometry has moved from a research tool to a routine component of preoperative planning for cataract and refractive surgery. Corneal higher-order aberration (HOA) profiles can contribute to assessing the optical quality and to evaluating the suitability of presbyopia-correcting intraocular lenses (IOLs) as one component of the broader clinical decision [1]. In addition, corneal HOA, such as coma-like aberrations, can be used to support the screening for early keratoconus before refractive procedures [2]. Because the posterior corneal surface contributes meaningfully to total corneal and ocular optical aberrations—particularly astigmatism and coma—instruments that measure only the anterior surface, such as Placido-disc topographers, are insufficient to fully characterize the anterior and posterior corneal surfaces [3,4].

Contemporary tomographic devices use several imaging techniques based on Scheimpflug photography, anterior segment optical coherence tomography (AS-OCT), or combinations of these technologies with Placido-disc topography. Rotating Scheimpflug camera-based systems, such as the Pentacam, Sirius, and Galilei, reconstruct anterior and posterior corneal elevation from a series of oblique slit-illuminated cross-sectional photographs; the Sirius and Galilei additionally combine Placido-disc topography [4,5,6,7]. AS-OCT-based systems, such as the Casia2, Anterion, and MS-39, use low-coherence interferometry with near-infrared light to resolve anterior-segment tissue interfaces in depth; the MS-39 incorporates OCT with Placido-disc topography [8,9,10,11]. Both platforms generate anterior and posterior elevation maps and calculate total corneal aberrations using Zernike polynomial decomposition [6,7,9,10]. In clinical studies, however, clinicians repeatedly observe that the same eye, measured within minutes on both platforms, can yield materially different HOA coefficients—an observation that has prompted a series of agreement studies over the past decade [12,13,14,15]. These differences may reflect not only the imaging principles but also reconstruction characteristics and device-specific acquisition [10,12,15].

The purpose of this review is threefold: (1) to describe how Scheimpflug imaging and AS-OCT reconstruct corneal surfaces and derive wavefront aberration data; (2) to synthesize the published literature comparing total corneal aberration measurements across devices incorporating Scheimpflug imaging, AS-OCT, and hybrid technologies; and (3) to analyze the specific optical and computational mechanisms that have been proposed to explain the observed discordance.

### Search Strategy

A structured search of PubMed and Google Scholar was performed from database inception to 8 September 2026, using combinations of the terms “corneal aberration,” “higher-order aberration,” “wavefront,” “Zernike,” “optical coherence tomography,” “OCT,” “Scheimpflug,” “agreement,” “Bland–Altman,” “intraclass correlation coefficient,” and “ICC”. We did not identify any dedicated review focused specifically on the agreement of total corneal aberration measurements across these devices. Available reviews address corneal topography and tomography technologies broadly [16,17] or focus on pachymetry, keratometry, and corneal elevation rather than wavefront aberration [18,19]. This review is intended to fill that gap and provide a framework for instrument selection, data interpretation, and future validation studies.

Studies were included for analysis if they (1) were original investigations directly comparing corneal tomographic devices based on AS-OCT, Scheimpflug imaging, or hybrid imaging systems, including comparisons between two Scheimpflug-based platforms; (2) reported total corneal wavefront or Zernike-derived aberration parameters; (3) included human eyes; (4) reported inter-device agreement statistics, such as Bland–Altman limits of agreement (LoA) or intraclass correlation coefficients (ICC); and (5) were published in peer-reviewed English-language journals. Animal or ex vivo studies, studies without quantitative agreement statistics, and conference abstracts without a full peer-reviewed publication were excluded. Representative studies meeting these criteria are summarized in Table 1. Table 1 is not intended to be exhaustive; the studies were selected to illustrate different device pairings, including OCT-based, Scheimpflug-based, and hybrid systems, and different study populations.

## 2. Optical Principles of Corneal Tomography

**Figure 1:**
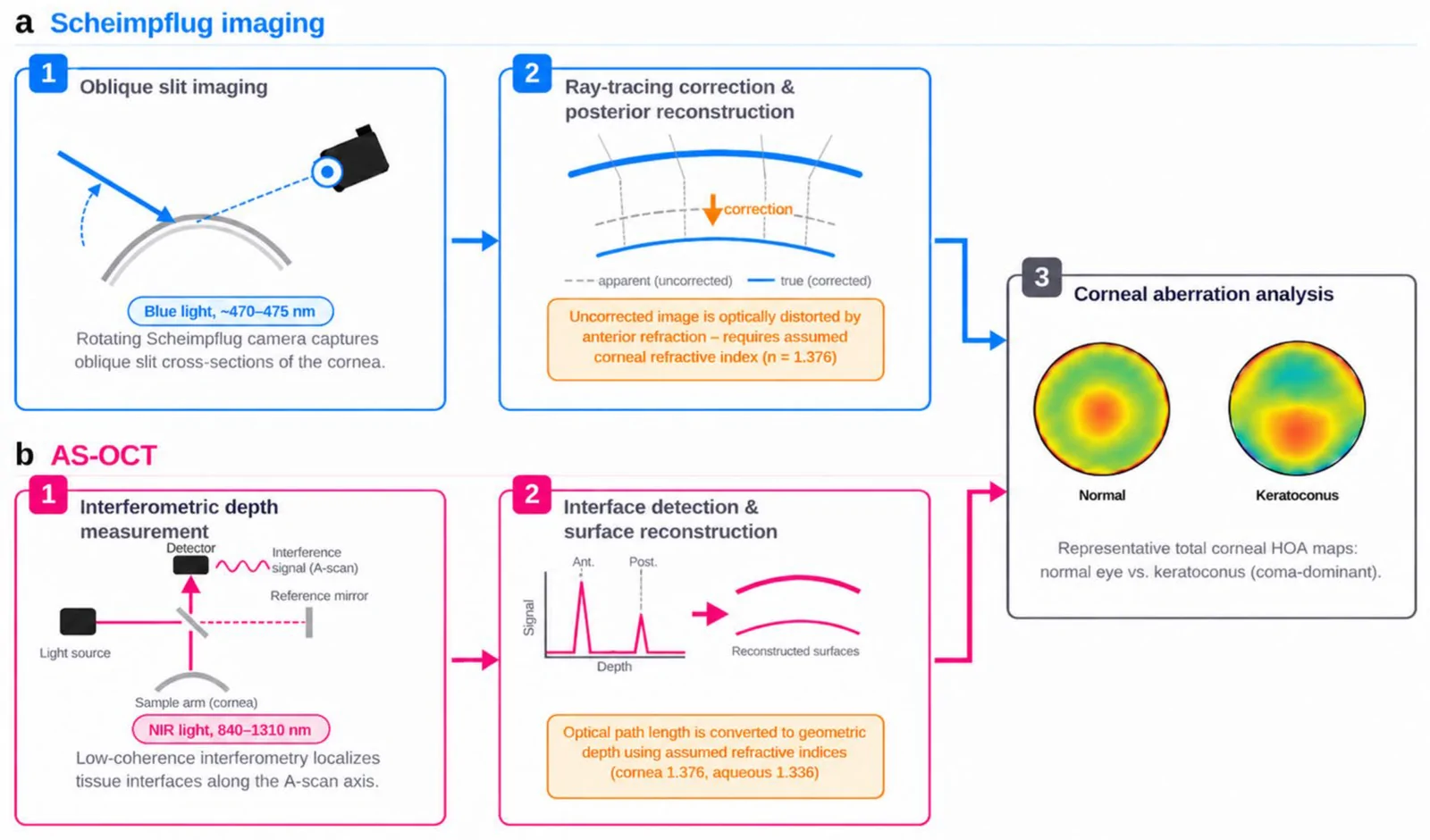
Schematic comparison of Scheimpflug imaging and anterior segment optical coherence tomography (AS-OCT) for total corneal aberration measurement. (**a**) Scheimpflug imaging reconstructs the anterior and posterior corneal surfaces from oblique slit images after optical correction. (**b**) AS-OCT detects corneal interfaces using low-coherence interferometry and reconstructs the corneal surfaces. Representative total corneal higher-order aberration wavefront maps are shown for a normal eye and a keratoconus eye.

Scheimpflug imaging and AS-OCT differ in how corneal interfaces are detected and reconstructed: Scheimpflug systems derive corneal surfaces from oblique slit images with optical distortion correction, whereas AS-OCT localizes tissue interfaces using low-coherence interferometric signals (Figure 1).

### 2.1. Scheimpflug Imaging

Scheimpflug photography exploits the Scheimpflug principle: when the object plane, lens plane, and image (sensor) plane of an optical system intersect along a common line rather than lying parallel to one another, a tilted object plane can be brought entirely into focus despite its extended depth of field [20]. In corneal tomographers, a slit beam illuminates a meridional cross-section of the anterior segment, while a camera oriented obliquely to the slit beam captures light scattered from tissue interfaces [20]. The Pentacam HR, for example, rotates a Scheimpflug camera around the visual axis and acquires 50 or 100 cross-sectional images in approximately 2 s, yielding up to 138,000 true elevation points for reconstruction of the three-dimensional corneal surface [21]. The Sirius combines a single rotating Scheimpflug camera with a Placido disk topographer, whereas the Galilei combines dual Scheimpflug cameras with a Placido disk [22].

A critical optical feature of Scheimpflug imaging is that the posterior corneal surface is viewed through the anterior surface: light reaching the camera from the posterior interface has already been refracted at the anterior corneal surface, so the raw image represents an optically distorted image of the true posterior surface [23]. Manufacturer software must therefore correct for refraction at the anterior corneal surface when reconstructing the true posterior surface position, using assumed corneal refractive indices and the simultaneously measured anterior surface geometry [24,25]. Because this process depends on assumed values, errors in the assumptions may propagate into posterior and total corneal wavefront aberration estimates [25,26]. Historical experience with the scanning-slit Orbscan system illustrates this vulnerability: inadequate correction of refractive distortion caused the posterior cornea to be localized too anteriorly, leading to underestimation of corneal thickness and apparent changes that could mimic postoperative ectasia [27,28,29,30].

### 2.2. Anterior Segment Optical Coherence Tomography

AS-OCT is based on low-coherence interferometry. A broadband or swept-wavelength near-infrared light source illuminates the eye, and light backscattered from tissue interfaces is combined with light from a reference arm; the resulting interference signal is used to localize reflective boundaries along the optical depth (A-scan) axis, either in the time domain or, in modern instruments, in the Fourier domain after spectral or wavelength-swept acquisition [31]. Swept-source OCT (SS-OCT) devices such as the Casia2 and Anterion use tunable, long-wavelength lasers operating at 1310 nm and approximately 1300 nm, respectively. These wavelengths provide deeper penetration with reduced sensitivity roll-off, enabling simultaneous imaging of the cornea, anterior chamber, and crystalline lens. The Casia2 acquires 50,000 A-scans per second over a 13 mm imaging depth, while the Anterion acquires 50,000 A-scans per second and obtains 65 radial B-scans, each comprising 256 A-scans, in under one second [32]. Spectral-domain (SD-OCT) devices such as the MS-39 use a shorter 840 nm light source, providing higher axial resolution (approximately 3.5 µm in tissue) but shallower penetration; the MS-39 is combined with Placido-disc anterior curvature data through a proprietary fusion algorithm [12].

Because OCT localizes each interface from interferometric optical-path measurements rather than by reconstructing oblique images, it is often described as less dependent on ray-tracing correction through the anterior surface than Scheimpflug imaging. This description is only partially accurate: OCT measures optical path length rather than physical distance, and converting the interferometric signal into geometric depth still requires assumed refractive indices for the cornea and aqueous humor (commonly 1.376 and 1.336, respectively, distinct from the traditional keratometric index of 1.3375 used in single-surface keratometry) [33]. Segmentation software must also correctly identify the anterior and posterior corneal boundaries from the A-scan intensity profile, and this process may be affected by image noise, motion artifacts, and reduced signal strength at oblique angles of incidence in the peripheral cornea [34]. Both modalities, therefore, share common vulnerabilities—dependence on an assumed refractive index and on automated boundary-detection software—but the specific optical pathways by which each modality is affected differ fundamentally. These differences may help explain why the resulting errors do not necessarily correspond or cancel across platforms.

## 3. Optical and Algorithmic Sources of Inter-Device Discordance

**Figure 2:**
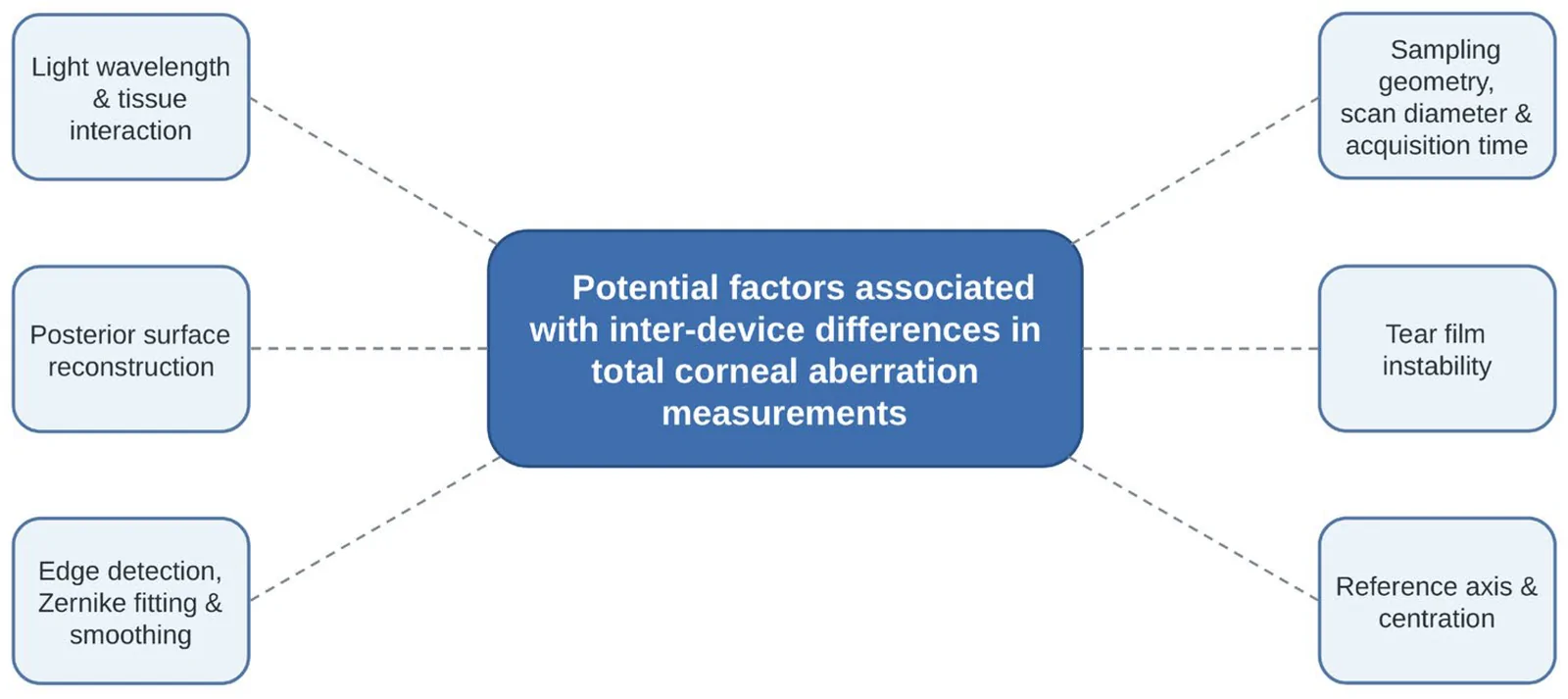
Potential factors associated with inter-device discordance in total corneal aberration measurements. Differences in light wavelength, posterior-surface reconstruction, image-processing algorithms, sampling geometry, tear film instability and reference-axis selection are considered possible contributors.

The potential factors associated with inter-device discordance are summarized in Figure 2. The mechanisms discussed below may reflect both modality-related differences in image acquisition and device-specific differences in reconstruction, processing, and analysis.

### 3.1. Light Source Wavelength and Light-Tissue Interaction

Scheimpflug systems typically use visible blue light with wavelengths of approximately 470–475 nm, which is subject to greater corneal light scattering and is more strongly attenuated by stromal haze or opacity than the near-infrared light with wavelengths of 840–1310 nm used by AS-OCT [22,32,35]. Because scattering and attenuation can influence the apparent position and contrast of the posterior corneal interface, corneas with even subclinical haze, scarring, or altered hydration may show larger inter-device discrepancies than optically pristine corneas. Inter-device differences in pachymetry and tomography have been reported in corneas with opacity, after crosslinking, and with cystinosis [35,36,37], suggesting that light source wavelength and light-tissue interaction can act as possible contributors to inter-device differences.

### 3.2. Posterior-Surface Reconstruction and Refractive-Index Assumptions

As described in Section 2, both technologies rely on assumed refractive indices and device-specific reconstruction algorithms to translate raw signals into physical posterior surface coordinates. Several agreement studies have reported that ICCs and LoA tend to be less favorable for posterior-surface and total corneal parameters than for anterior-surface parameters in several device pairs, and posterior corneal aberration parameters have also shown lower within-device precision [10,14,38,39]. Refractive-index assumptions, segmentation, and posterior-surface reconstruction may collectively contribute to this pattern.

### 3.3. Edge Detection, Zernike Fitting, and Smoothing Algorithms

Raw tomographic data from either modality consist of a finite set of two-dimensional cross-sections that must be converted into a continuous three-dimensional surface through boundary detection, interpolation, surface fitting, and smoothing—steps that are largely proprietary and may differ among manufacturers [40]. Using a rabbit corneal model, Baraya and colleagues showed that Pentacam anterior and posterior surface data required 12th- and 10th-order Zernike polynomial fitting, respectively, with additional smoothing, to replicate manufacturer-reported curvature maps, and that this fitting process itself introduced measurable errors in axial and tangential curvature [40]. Because HOA coefficients are derived through Zernike decomposition, differences in fitting order, smoothing, outlier handling, and other reconstruction procedures between manufacturers may contribute to systematic inter-device differences even when the acquired elevation data are similar [41]. This mechanism is supported indirectly by studies showing discordance even between Scheimpflug-based platforms using conceptually similar optical principles, such as the Pentacam and Galilei, suggesting that device-specific reconstruction software, in addition to optical design, can contribute to disagreement [15].

### 3.4. Sampling Geometry, Scan Diameter, and Acquisition Time

Scheimpflug tomographers typically acquire 25–50 meridional cross-sections over approximately 1–2 s, whereas modern SS-OCT devices acquire 65 or more radial B-scans, each comprising hundreds of A-scans, in approximately one second or less [9,12,23]. Denser, faster sampling can reduce susceptibility to microsaccades, tear-film disruption, and blink-related artifacts, potentially affecting higher-order, spatially localized terms such as coma and trefoil [10,42]. Differences in maximum scan diameter, commonly 8–9 mm, and in the corneal zone used for Zernike fitting, e.g., 4 mm versus 6 mm optical zone, may further affect HOA magnitude, since higher-order Zernike coefficients are highly sensitive to the exact pupil or analysis diameter and to edge effects of the sampled region [14,43]. Therefore, before concluding that two devices disagree, the analysis diameter and reference center should be matched; Zernike coefficients obtained over a larger diameter can be mathematically rescaled to a smaller diameter, but not the reverse [43].

### 3.5. Tear Film Instability

Tear film instability can generate higher spatial-frequency aberrations, which can reduce the performance and accuracy of Zernike-based modeling [44]. Central corneal thickness measurements obtained using the Pentacam showed lower repeatability in dry eyes than in healthy eyes, which improved after use of artificial tears [45]. However, measurements obtained using the Galilei showed high repeatability in mild dry eyes, although anterior axial curvature within the central 3-mm zone showed variability [46]. Because measurements are acquired sequentially, changes in the tear film may cause the two devices to capture different anterior corneal surfaces. Therefore, tear film instability may contribute to inter-device HOA discordance; however, the available evidence is indirect, and direct studies on agreement between Scheimpflug and AS-OCT measurements in dry eye remain limited.

### 3.6. Reference Axis and Centration

Wavefront decomposition requires a defined reference axis, and manufacturers differ in whether they center the analysis on the pupil center, the corneal vertex, or the visual axis. Because third-order aberrations such as coma and trefoil are sensitive to decentration relative to the chosen reference axis, differences in axis definition or in the software’s determination of the corneal apex can contribute to meaningful differences in reported values between devices measuring the same eye [3,47]. Poor inter-device agreement has also been reported for angle kappa and pupil-center offset measurements, suggesting that centration methodology is another possible contributor to aberrometric discordance [14].

## 4. Summary of Comparative Studies

Table 1 summarizes representative studies directly comparing corneal aberration parameters across available tomographic devices, including OCT-based, Scheimpflug-based, and hybrid systems. These devices differ in acquisition geometry, Placido integration, analysis zone, reference center or axis, and reconstruction methods as well as imaging modality; therefore, observed differences may reflect many inter-device variables rather than the imaging modality alone [10,12,13,14,15]. Across studies, agreement varied by the paired device, study population, analysis diameter, reference center or axis, and aberration parameters [10,12,13,14,15]. Studies involving the Placido-hybrid MS-39 reported relatively small mean differences for multiple corneal aberration parameters in comparisons with the Sirius or Anterion [10,12]. The comparison between Anterion and MS-39 showed a mean difference of 0.03 µm for total HOA RMS, with 95% LoA ranging from −0.22 to 0.28 µm [12]. Although the mean difference was small (0.03 µm), the 95% LoA spanned 0.50 µm, indicating that individual paired measurements could differ by approximately 0.25 µm in either direction relative to the mean difference. Because no clinically acceptable tolerance for total corneal HOA RMS has been established, these findings do not establish interchangeability between the two devices. Other studies comparing Anterion with Pentacam HR [13,14] and two Scheimpflug-based platforms [15] reported variable agreement across total HOA RMS and individual HOA parameters. These findings suggest that inter-device agreement depends on the specific device pair and aberration parameter, although the contributions of individual device characteristics remain uncertain.

Notably, the reference center or axis used for calculating aberration measurements was not clearly reported in several studies summarized in Table 1. This is relevant because Zernike-derived corneal aberration coefficients may vary according to the reference center used, such as the corneal vertex or the pupil center [48]. Whole-eye wavefront aberrations are generally referenced to the pupil center and the line of sight, making the reference center or axis particularly important when interpreting corneal aberrations [49].

## 5. Clinical Implications

### 5.1. Cataract Surgery and Intraocular Lens Selection

Quantitative ocular imaging and optical simulation of postoperative outcomes can help guide the selection of optimal treatments in modern cataract surgery [50]. Because modern IOLs differ in their control of spherical and chromatic aberrations and mechanisms for extending the range of focus, precise characterization of preoperative ocular optics can be fundamental to IOL selection [51].

Total corneal astigmatism is useful in toric IOL selection, whereas total corneal spherical aberration is relevant in aspheric IOL selection [1,52]. Toric IOL cylinder power and axis are determined primarily by corneal astigmatism, measured or estimated with formula-specific posterior corneal adjustments, whereas HOA coefficients are not routinely used as inputs in toric IOL calculations [4]. Given that total corneal power and astigmatism measurements have shown statistically significant, though often modest, differences between tomographic and biometric devices, particularly for posterior and total rather than anterior-only astigmatism, clinicians should be cautious when measurements obtained from different devices are used interchangeably in IOL planning [53,54].

When simultaneous-vision IOLs providing a partial or full range of field are considered, residual corneal HOAs may be clinically relevant, since coma, trefoil, and total HOA may be associated with visual performance across the defocus range [55,56]. However, corneal HOAs represent only one component of this decision, alongside other ocular and patient factors. For one diffractive multifocal IOL, total corneal HOA below 0.3 µm, coma and trefoil below 0.2 µm, and spherical aberration below 0.1 µm at a 4-mm diameter were considered safe [55]. Inter-device differences in corneal HOA measurements may influence the interpretation of values near such thresholds [12,14]. However, these differences can vary depending on the aberration parameter, analysis diameter, and device pair. Whether such differences change IOL selection or clinical outcomes has not been directly studied.

### 5.2. Keratoconus and Ectasia Screening

Corneal tomography, including anterior and posterior elevation and pachymetric measurements, is commonly used to detect and evaluate keratoconus and corneal ectasia [57]. Posterior corneal changes can be useful in identifying early or forme fruste keratoconus [9,57]. However, measurements of elevation and other parameters can be different between devices, including AS-OCT and Scheimpflug systems [39].

Corneal HOAs, particularly coma-like aberrations, are increased in keratoconus and can help detect corneal optical asymmetry, including subclinical disease [58]. Because individual HOA measurements show variable agreement across contemporary devices [14], it may be helpful to use the same device for clinical assessment and longitudinal follow-up.

### 5.3. Refractive Surgery Planning

Coma and trefoil are of particular interest in irregular astigmatism and post-refractive surgery evaluation. In topography-guided or wavefront-guided surgery, treatment planning may rely on these aberrations; Gim et al. reported 95% LoA of −0.43 to 0.32 µm for horizontal trefoil at 6 mm [13] and Pérez-Bartolomé et al. reported −0.408 to 0.316 µm for vertical coma at 6 mm [14]. For this reason, measurements obtained from different devices should be interpreted with caution [13,14]. For serial assessment before and after refractive surgery, use of the same measurement system may provide a more internally consistent comparison.

## 6. Limitations of Current Evidence and Future Directions

Most published comparisons are cross-sectional agreement studies using Bland–Altman analysis and ICC in heterogeneous clinical cohorts. These studies can indicate the presence of inter-device disagreement but cannot establish whether the observed differences are due to optical factors, such as wavelength and refractive-index assumptions, or device-specific acquisition geometry and software, including surface reconstruction, Zernike fitting, and smoothing algorithms. In addition, these studies are heterogeneous in terms of population, analysis diameter, reference center or axis, acquisition protocol, and the aberration parameters. These discrepancies in methods can limit precise comparison between studies. In addition, it is difficult to generalize the disagreement to other devices or patient groups.

Therefore, future studies should use more standardized acquisition protocols and reporting parameters, including the analysis diameter, reference center or axis, pupil conditions if relevant, and device-specific settings. Prospective studies with larger populations and diverse eye conditions, such as keratoconus and post-refractive surgery corneas, can help explain the effect of clinical condition on inter-device agreement. Model-eye and artificial cornea validation studies, in which the true surface geometry is known, could allow direct quantification of instrument-specific bias independent of biological variability. Ray-tracing simulations that vary only the assumed refractive index or Zernike fitting order, holding true surface shape constant, may similarly isolate the algorithmic contribution described in Section 3.3. Beyond statistical agreement, future studies should determine whether inter-device differences are clinically meaningful and whether they affect IOL selection, surgical planning, or postoperative assessment. Greater manufacturer transparency regarding reconstruction algorithms, and the development of a device-agnostic reference standard for total corneal HOA, would strengthen the evidence base and support more confident clinical use of total corneal aberration data across platforms.

## 7. Conclusions

Scheimpflug imaging and AS-OCT rest on different optical principles—photographic capture of an obliquely oriented, refraction-distorted image versus interferometric depth-ranging of backscattered near-infrared light—yet both depend on device-specific acquisition, refractive-index assumptions, and proprietary surface-reconstruction software to generate a total corneal wavefront map. Inter-device differences should be interpreted in the context of not only the imaging modality but also the measurement system, including device architecture and software. Agreement in the published literature varies by device pair, parameter, and analysis diameter. Mean inter-device differences were generally small, whereas the 95% LoA for total HOA RMS spanned approximately 0.33–0.67 µm at 5–6 mm, and comparably wide intervals were reported for individual terms such as horizontal trefoil and vertical coma at 6 mm. Posterior-surface parameters showed larger mean differences and lower precision in several studies. This pattern suggests that posterior-surface reconstruction, Zernike-fitting and smoothing algorithms, sampling geometry, tear film instability, and reference-axis selection may collectively contribute to inter-device discordance. However, inter-device statistical disagreement does not necessarily translate into clinically meaningful differences; whether this discordance affects IOL selection, surgical planning, or clinical outcomes needs further research. Clinicians should treat total corneal HOA values, particularly posterior-surface-derived and higher-order terms, as device-specific rather than universally interchangeable. Whenever feasible, using the same device for longitudinal comparison can be recommended to minimize measurement-related variability.

## Figures and Tables

**Table 1:** Summary of comparative studies evaluating inter-device agreement in total corneal aberration measurements.

**Study**	**Devices**	**Population (n)**	**Anterior-Surface Data Source**	**Analysis Zone**	**Reference Center/Axis**	**Agreement Findings**
Ning et al., 2023 [10]	MS-39 vs. Sirius	56 eyes after SMILE	MS-39: SD-OCT + Placido Sirius: Scheimpflug + Placido	6 mm	NR	Mean differences (µm): −0.10 to 0.04; largest absolute mean differences in posterior total RMS (−0.10) and posterior HOA RMS (−0.07) 95% LoA (µm): total RMS, −0.13 to 0.21; total HOA RMS, −0.15 to 0.18; coma, −0.15 to 0.22; trefoil, −0.16 to 0.15; spherical aberration, −0.08 to 0.09 ICC: NR
Georgiev et al., 2023 [12]	Anterion vs. MS-39	103 eyes of cataract surgery candidates	Anterion: SS-OCT MS-39: SD-OCT + Placido	5 mm	Pupil center	Absolute mean differences ≤ 0.05 µm for all parameters 95% LoA (µm): total HOA RMS, −0.22 to 0.28; vertical coma, −0.33 to 0.25; horizontal coma, −0.20 to 0.22; spherical aberration, −0.11 to 0.08 ICC: NR
Gim et al., 2021 [13]	Anterion vs. Pentacam HR	53 healthy eyes	Anterion: SS-OCT Pentacam HR: Scheimpflug	6 mm	Corneal vertex (Anterion); NR (Pentacam HR)	95% LoA (µm): total HOA RMS, −0.24 to 0.30; vertical coma, −0.31 to 0.23; horizontal trefoil, −0.43 to 0.32; spherical aberration, −0.09 to 0.24; fifth-order RMS, −0.21 to 0.10; sixth-order RMS, −0.17 to 0.03 ICC: NR
Pérez-Bartolomé et al., 2022 [14]	Anterion vs. Pentacam HR	50 healthy eyes	Anterion: SS-OCT Pentacam HR: Scheimpflug	4 & 6 mm	Corneal vertex (Anterion); NR (Pentacam HR)	Total HOA RMS (4/6 mm): ICC 0.718 (95% CI, 0.345–0.848)/0.742 (0.200–0.814); 95% LoA (µm) −0.151 to 0.111/−0.366 to 0.308 Vertical coma (4/6 mm): ICC 0.834 (95% CI, 0.684–0.913)/0.840 (0.669–0.923); 95% LoA (µm) −0.187 to 0.127/−0.408 to 0.316
Piccinini et al., 2019 [15]	Pentacam HR vs. Galilei G4	105 healthy eyes	Pentacam HR: Scheimpflug Galilei G4: dual Scheimpflug + Placido	6 mm	NR	95% LoA (µm): total RMS, −0.536 to 1.081; horizontal coma, −0.125 to 0.427; vertical coma, −0.328 to 0.217; spherical aberration, −0.159 to 0.159 ICC: NR

Abbreviations: CI, confidence interval; HOA, higher-order aberration; ICC, intraclass correlation coefficient; LoA, limits of agreement; NR, not reported; RMS, root mean square; SD-OCT, spectral-domain optical coherence tomography; SMILE, small-incision lenticule extraction; SS-OCT, swept-source optical coherence tomography. For Pérez-Bartolomé et al., ICCs were calculated using a two-way mixed-effects model for absolute agreement. In Gim et al., Anterion reported Zernike coefficients to one decimal place, and Pentacam HR values were rounded to the same precision for comparison. This limited numerical resolution may have contributed to the wider 95% LoA for fifth- and sixth-order RMS values.

## Data Availability

No new data were created or analyzed in this study. Data sharing is not applicable to this article.

## Abbreviations

The following abbreviations are used in this manuscript:

AS-OCT	Anterior segment optical coherence tomography
HOA	Higher-order aberration
ICC	Intraclass correlation coefficient
IOL	Intraocular lens
LoA	Limits of agreement
OCT	Optical coherence tomography
RMS	Root mean square
SD-OCT	Spectral-domain optical coherence tomography
SS-OCT	Swept-source optical coherence tomography
